# Endocrine Complications in Children and Adolescents With Non-Central Nervous System Solid Tumors

**DOI:** 10.3389/fendo.2021.610730

**Published:** 2021-03-17

**Authors:** Yena Lee, Juhee Shin, Yunha Choi, Hyery Kim, Kyung-Nam Koh, Ho Joon Im, Han-Wook Yoo, Jin-Ho Choi

**Affiliations:** Department of Pediatrics, Asan Medical Center Children’s Hospital, University of Ulsan College of Medicine, Seoul, South Korea

**Keywords:** cancer survivors, child, endocrine system disease, risk factors, alkylating agents, hematopoietic stem cell transplantation, solid tumors

## Abstract

**Background:**

Due to remarkable progress in cancer treatment, endocrine complications are now the major medical issues facing childhood cancer survivors. Although non-central nervous system solid tumors (NCSTs) account for approximately 40% of all pediatric cancers, there have been few studies on endocrine complications associated with NCSTs. This study investigated endocrinopathies following the treatment of pediatric NCSTs.

**Design and setting:**

Retrospective study in a single academic center.

**Methods:**

This study analyzed 253 survivors of childhood NCSTs who were diagnosed between January of 2000 and December of 2018. The medical charts were reviewed regarding the frequency of endocrinopathies and treatment modalities. The hazard ratios were assessed by multivariable Cox regression analysis. The final height-SDS were analyzed by multivariable linear regression analysis.

**Results:**

There were 76 patients (30%) that developed at least one endocrine complication. Forty-four patients (17.4%) experienced endocrine complications within five years of their cancer diagnosis. The most common endocrine complication was growth failure (n = 35), followed by obesity (n = 18), and primary gonadal failure (n = 16). High cumulative doses of alkylating agents increased the risk of developing at least one endocrine complication. Hematopoietic stem cell transplantation was an important risk factor for primary gonadal failure.

**Conclusions:**

This study described the comprehensive endocrine outcomes, including growth failure, obesity, primary gonadal failure, primary hypothyroidism, dyslipidemia, and osteoporosis, following the treatment of childhood NCSTs. As endocrinopathies occurred within five years of primary tumor diagnosis, surveillance for endocrine dysfunction is required for early intervention and management.

## Introduction

Pediatric non-central nervous system solid tumors (NCSTs) are a heterogeneous group of non-hematologic, extracranial cancers including neuroblastoma, soft tissue sarcoma, bone tumors, retinoblastoma, hepatoblastoma, germ cell tumors, and various other rare, solid organ tumors that predominantly occur during childhood (1). Multi-modality therapy, including surgical resection, chemotherapy, radiotherapy (RT), and autologous hematopoietic stem cell transplantation (HSCT), is required to manage children with NCSTs.

With the remarkable progress in cancer treatment, five-year childhood cancer survival rates have dramatically improved to approximately 80% in developed countries (2). With increases in survival, the late effects of cancer therapy have become a major medical issue of childhood cancer survivors. The majority of these survivors experience at least one non-endocrine or endocrine complication (3, 4). Endocrine disturbances are the most frequent complications in childhood cancer survivors, including hematologic malignancies, brain tumors, and sarcomas, with more than 50% of cancer survivors experiencing at least one hormonal disorder during their lifetime (5).

Generally, childhood survivors of acute lymphocytic leukemia achieve a normal final height with a body mass index (BMI) within the normal range (6, 7). Growth hormone deficiency (GHD) is the most common hypothalamic-pituitary disorder observed in survivors of brain tumors and those whose hypothalamic-pituitary region has been exposed to RT (8, 9). The prevalence of combined pituitary hormone deficiency has been found to be higher in patients with sellar and suprasellar tumors than in those with tumors in other locations (10).

Although NCSTs account for approximately 40% of all pediatric cancers (11), a few studies have examined their endocrine outcomes. Endocrinopathies after childhood cancer treatments, including growth failure, gonadal dysfunction, primary hypothyroidism, obesity, and disrupted bone heath, have been investigated in hematologic malignancies (12) and brain tumors (10, 13). Previous data on NCSTs were based on disease-specific cohorts. Female survivors of Wilms tumor who underwent abdominal radiation were reported to have poor fertility outcomes (14). Impaired fertility was demonstrated in Ewing sarcoma survivors as well (15). Pituitary dysfunction, after the treatment of head and neck rhabdomyosarcoma, has also been identified (16). In another study, hypothyroidism and gonadal dysfunction were the most frequent endocrine complications after the treatment of neuroblastoma (17).

To date, only one study included various NCSTs in a comprehensive analysis of endocrine outcomes (18). Therefore, this study investigated endocrine complications of pediatric patients with NCSTs and analyzed the risk factors related to endocrine complications and determinants of growth outcomes.

## Subjects and Methods

### Subjects

**Figure 1:**
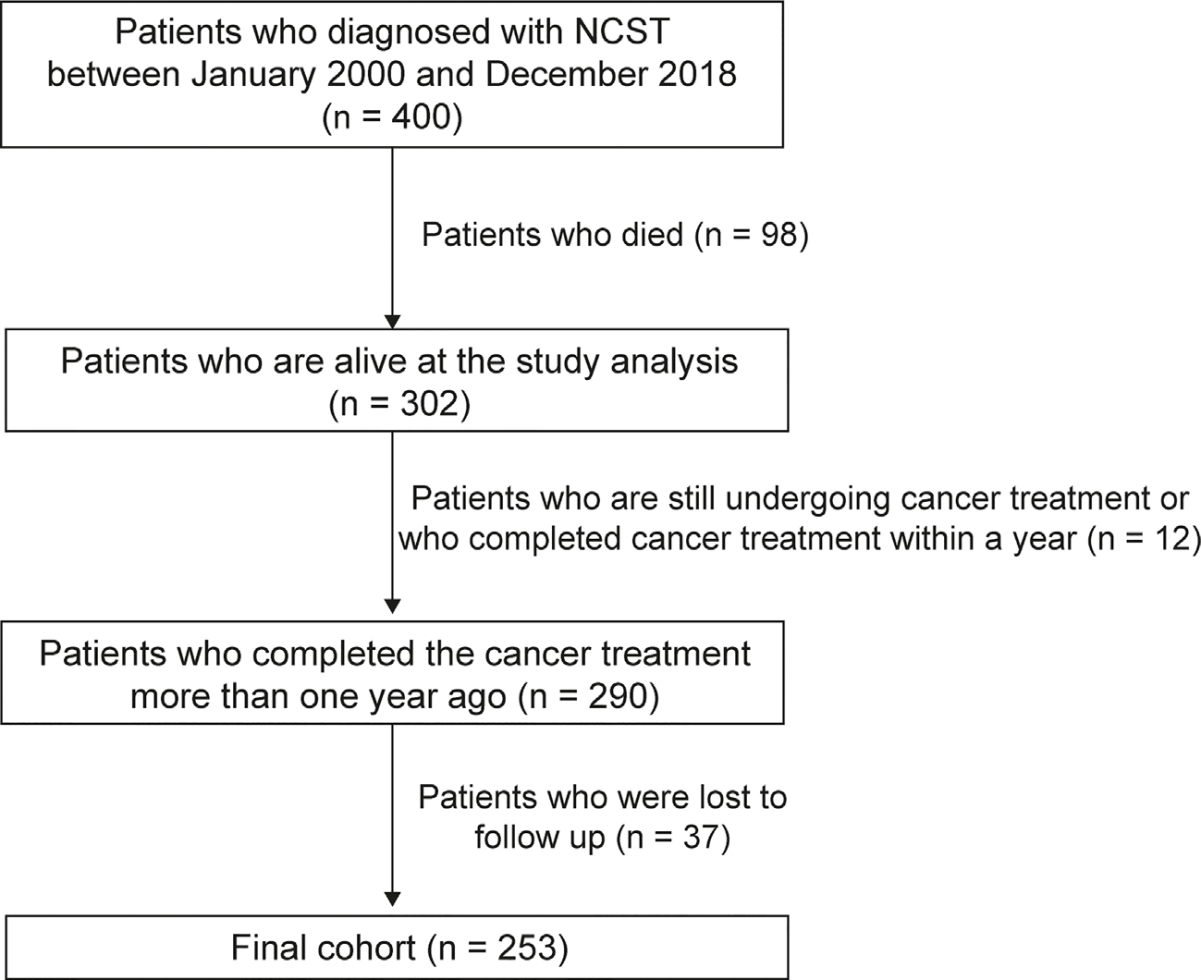
Diagram for study population.

A total of 400 patients were diagnosed with NCSTs before the age of 18 years at the Department of Pediatrics in Asan Medical Center Children’s Hospital between January of 2000 and December of 2018. Inclusion criteria for this study were: 1) patients diagnosed with NCSTs between January of 2000 and December of 2018; 2) patients younger than 18 years of age at primary tumor diagnosis; 3) patients alive at the time of the study analysis, and 4) those who completed the cancer therapy more than one year previously. The treatment endpoint was defined as the completion of treatment for a primary, metastatic, or relapsed cancer. Patients who were lost to follow-up during the observational period were excluded (**Figure 1**). Ultimately, 253 survivors were included in this study after the exclusion of 98 patients who died during the follow-up period, a further 37 patients were lost to follow-up, and 12 patients who did not fulfill the inclusion criteria.

Clinical characteristics and endocrine complications were analyzed by a retrospective medical chart review. This study was approved by the Institutional Review Board of Asan Medical Center, Seoul, Korea (IRB number 2020-1146).

### Treatment Modalities and Alkylating Agent Dose (AAD) Scores of Each Patient

Treatment modalities, including operation (OP), chemotherapy, RT, autologous and allogeneic HSCT, and ^131^I-metaiodobenzylguanidine (^131^I-MIBG) therapy were reviewed. The cumulative dose and location of RT were documented. The cumulative dose (mg/m^2^) of alkylating agents (busulfan, cyclophosphamide, ifosfamide, melphalan, and thiotepa) was assessed by an alkylating agent dose (AAD) score. The dose distribution for each agent was divided into tertiles. A score of zero was given to patients who did not get any alkylating agent. A score of one was assigned if the patient’s dose was within the first tertile, a score of two was assigned if it was within the second tertile, and a score of three was assigned if it was within the third tertile. The scores of each agent for an individual patient were summed, the result being the summed AAD score (19, 20).

### Endocrine Complications of Childhood Survivors With Non-Central Nervous System Solid Tumors (NCSTs)

Short stature was defined as the height-SDS below -2.0 SDS for the age- and gender-matched normative data from Korean references (21).Growth deceleration was defined as growth velocity below the fifth percentile for age and gender (e.g., <5 cm/year after the age of five years) or a drop in height across two or more percentiles on the growth chart before closure of the growth plate (22, 23). Insulin-like growth factor-1 (IGF-1) SDSs were calculated based on the reference values of Korean children and adolescents (24). Obesity was a BMI above 95 percentile for the age- and gender-matched reference data (21). A diagnosis of hypergonadotropic hypogonadism was based on elevated luteinizing hormone (LH) and follicle-stimulating hormone (FSH) levels with low testosterone or estradiol levels (25). Vitamin D deficiency was a level of 25-hydroxyvitamin D3 (25OHD3) less than 20 ng/mL (26). Osteoporosis was diagnosed in patients with a bone mineral density (BMD) *Z*-score below -2.0 on a dual-energy X-ray absorptiometry (DEXA) scan (Lunar Corp., Madison, WI, USA). BMD Z-scores were adjusted by age- and gender-matched standards for Korean children (27, 28). As the Korean reference data for children aged ≥10 years were measured by the Hologic system, the BMD data were converted with the following formula: lumbar spine BMD by Hologic system = 0.837 × lumbar spine BMD by Lunar system + 0.021; and femoral neck BMD by Hologic system = 0.913 × femoral neck BMD by Lunar system - 0.080 (29).

Survivors of NCSTs were followed-up annually at pediatric endocrine and/or hematology/oncology clinics. The following parameters were annually assessed annually: height, weight, BMI, Tanner stage, bone age, lipid profiles, serum IGF-1 and IGF binding protein 3 (IGFBP-3) levels, and thyroid function tests (thyroid-stimulating hormone [TSH], free T4). Serum LH, FSH, testosterone (male) or estradiol (female) levels were measured after pubertal age. DEXA was performed consecutively in select patients, such as adult patients with primary hypogonadism or those treated with long-term corticosteroids. The follow-up and treatment protocols were based on the previous guidelines (30–32).

### Statistical Analysis

Statistical analyses were performed using IBM SPSS Statistics for Windows version 21.0 (IBM Co., Armonk, NY, USA). Multivariable analyses were performed using Cox proportional hazards model to identify hazard ratio (HR) of endocrine complications according to the treatment modalities of NCST. Time variables, defined as duration (years) from the primary cancer diagnosis to the date of the final visit or diagnosis of the endocrine complication, were used for the Cox regression analyses. Multiple variables were evaluated by employing backward stepwise regression analysis. Kaplan-Meier curves were generated to illustrate the risk-associated cumulative incidence of endocrine dysfunction. The differences in height-SDS between initial and final value was analyzed by a paired t-test. Factors affecting final height-SDS were assessed by univariable and multivariable linear regression analysis in survivors who reached their final height (age ≥ 18 years). The Mann-Whitney test was used to compare the age at HSCT between patients with and without hypogonadism. *p*-value < 0.05 was considered statistically significant.

## Results

### Patient Characteristics and Treatment Modalities

The mean age at diagnosis of NCSTs was 4.9 ± 4.9 years (range 0–16 years). The mean age at the outcome assessment was 13.9 ± 6.7 years (range 1.5–29 years). The mean follow-up duration was 8.95 ± 4.6 years (range 1.3–22 years). Sixty-four percent of the patients were observed ≤10 years, and 36% of the study subjects continued to attend annual follow-up visits more than 10 years after their primary NCST diagnosis. Primary diagnosis included neuroblastoma (25.7%), hepatoblastoma (21.3%), osteosarcoma (16.6%), Wilms tumor (15.4%), rhabdomyosarcoma (10.7%), Ewing sarcoma (9.9%), and ectomesenchymoma (0.4%) (**Table 1**). Treatment modalities are summarized in **Table 2**. Four patients with genetic syndromes were included: Beckwith–Wiedemann syndrome (BWS, n = 2), Noonan syndrome (NS, n =1), and WAGR syndrome (n = 1).

**Table 1:** Characteristics of childhood solid tumor survivors.

Characteristics		N	%
Gender	Female	108	42.7
	Male	145	57.3
Age at diagnosis (years)	0 to 4 years	150	59.3
	5 to 9 years	43	17.0
	10 to 14 years	48	19.0
	≥15 years	12	4.7
Age at study (years)	0 to 10 years	92	36.2
	11 to 20 years	114	44.9
	20 to 30 years	47	18.5
Diagnosis	Neuroblastoma	65	25.7
	Hepatoblastoma	54	21.3
	Osteosarcoma	42	16.6
	Wilms tumor	39	15.4
	Rhabdomyosarcoma	27	10.7
	Ewing sarcoma	25	9.9
	Ectomesenchymoma	1	0.4
Treatment duration (months)	0 to 12 months	190	75.1
	13 to 24 months	47	18.6
	25 to 36 months	8	3.2
	≥36 months	8	3.2
Follow-up duration (years)	0 to 5 years	67	26.5
	6 to 10 years	95	37.5
	11 to 15 years	64	25.3
	16 to 20 years	26	10.3
	≥21 years	1	0.4
Cancer-free survival (years)	0 to 5 years	81	32.0
	6 to 10 years	102	40.3
	11 to 15 years	52	20.6
	≥16 years	18	7.1

**Table 2:** Treatment modalities used in patients with childhood solid tumors.

Treatment		Number	%
Treatment modalities	Operation only	11	41.9
	Chemotherapy only	6	2.4
	OP + chemotherapy	148	58.5
	OP + RT	4	1.6
	OP + chemotherapy + RT	36	14.2
	OP + chemotherapy + HSCT	16	6.3
	Chemotherapy + RT + HSCT	1	0.4
	OP + chemotherapy + RT + HSCT	31	12.3
Summed AAD score	0	106	41.9
	1–4	64	25.3
	5–8	35	13.8
	9–12	12	4.7
	No data	36	14.2
Location of RT	Head and neck	12	4.7
	Chest	8	3.2
	Abdomen and pelvis	45	17.8
	Extremities	3	1.2
	Chest and extremities	2	0.8
	Total body irradiation	2	0.8
Cumulative dose of RT	None	181	71.5
	0 to < 30 Gy	40	15.8
	≥30 Gy	31	12.3
	No data	1	0.4
HSCT	Autologous	45	17.8
	Allogenic	3	1.2
^131^I-MIBG therapy	Yes	8	3.2

OP, operation; RT, radiotherapy; HSCT, hematopoietic stem cell transplantation; AAD, alkylating agent dose; ^131^I-MIBG, ^131^I -metaiodobenzylguanidine.

### Endocrine Complications Associated With NCST Treatment

**Figure 2:**
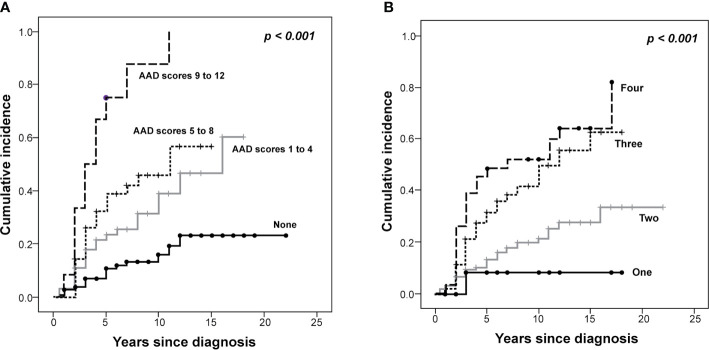
The Kaplan–Meier curves of the cumulative incidence of endocrine complications in survivors of childhood solid tumors. The cumulative incidence of endocrine complications is demonstrated according to the **(A)** alkylating agent dose (AAD) scores and the **(B)** number of treatment modalities (*P* < 0.001).

Endocrine complications were found in 76 patients (Male (M):Female (F) = 30:46) (**Table 3**). Among them, 44 patients (17.4%) manifested endocrine complications within five years of NCST diagnosis. The risk of endocrine complications increased according to the summed AAD score (**Table 4**). HSCT is an important risk factor for developing at least one endocrine complication (HR = 2.44, *p* = 0.024). The cumulative incidence of any endocrine complication increased with a higher summed AAD score and a greater number of treatment modalities (*p* < 0.001, **Figure 2**).

**Table 3:** The endocrine complications in survivors of childhood solid tumors.

		Numbers	%	Timing of complications		Time from initial cancer diagnosis to the diagnosis of endocrinopathies (years, mean ± SD)
				During Tx. (numbers)	After Tx. (numbers)	
Endocrine complications	Yes	76	30.0	–	–	5.0 ± 3.9
	No	177	70.0	–	–	–
Specific diagnosis	Short stature	37	14.6	–	–	–
	Growth failure	35	13.8	6	29	3.9 ± 2.9
	Obesity	18	7.1	0	18	6.7 ± 4.3
	Hypergonadotropic hypogonadism	16	12.1*	2	14	5.4 ± 3.8
	Primary hypothyroidism	12	4.7	1	11	4.1 ± 2.9
	Dyslipidemia	8	3.2	1	7	5.4 ± 5.0
	Vitamin D deficiency	7	2.8	5	2	2.4 ± 1.7
	Osteoporosis	3	1.2	0	3	2.3 ± 0.6
	Vitamin D deficiency rickets	2	0.8	2	0	1.3 ± 0.8
	Central precocious puberty	2	0.8	0	2	4.0 ± 1.4
	Secondary thyroid cancer	2	0.8	0	2	8.5 ± 5.0
	Hypoparathyroidism	1	0.4	0	1	5
	Adrenal insufficiency	1	0.4	0	1	0.5
	Multiple pituitary hormone deficiencies	1	0.4	0	1	2
	Isolated GH deficiency	1	0.4	0	1	2
	Hypophosphatemic rickets	1	0.4	1	0	4
	Hypothalamic amenorrhea	1	0.4	0	1	3

Tx, cancer treatment; SD, standard deviation.

*The incidence of gonadal failure was calculated in patients who reached post-pubertal age (F ≥13, M ≥14).

**Table 4:** Multivariable Cox proportional hazard analysis of endocrine complications following solid tumor treatment in childhood cancer survivors.

	Any endocrine complication			Growth failure			Obesity			Gonadal failure*		
	HR	95% CI	*p* value	HR	95% CI	*p* value	HR	95% CI	*p* value	HR	95% CI	*p* value
Gender												
Male	1.0			1.0			1.0			1.0		
Female	3.12	1.81–5.35	<0.001	2.70	1.25–5.82	0.011	3.22	1.02–10.17	0.047	30.31	4.20–218.47	0.001
Treatment duration												
0 to 12 months				1.0								
13 to 24 months				2.06	0.78–5.39	0.143						
25 to 36 months				6.36	1.58–25.57	0.009						
> 36 months				0.81	0.10–6.75	0.842						
Summed AAD score												
0 (None)	1.0											
1 to 4	2.50	1.26–4.97	0.009									
5 to 8	2.83	1.15–6.99	0.024									
9 to 12	4.72	1.55–14.40	0.006									
HSCT												
Not done	1.0			1.0			1.0			1.0		
Yes	2.44	1.13–5.30	0.024	2.58	1.03–6.50	0.044	3.30	0.96–11.34	0.058	46.39	8.25–260.69	<0.001
Radiotherapy												
Not done				1.0						1.0		
Yes				5.34	2.12–13.48	<0.001				5.56	1.33–23.31	0.019

Adjusted for gender, age at cancer diagnosis, treatment durations, treatment modalities (RT, HSCT), summed AAD scores, cumulative dose of RT, head and neck RT, and abdominal RT.

*Analysis of gonadal failure was performed only in patients who reached puberty age (F ≥13, M ≥14).

HR, hazard ratio; CI, confidence interval; AAD, alkylating agent dose; HSCT, hematopoietic stem cell transplantation; RT, radiotherapy.

#### 1. Short Stature and Growth Failure

Thirty-seven patients (14.6%, M:F = 15:22) had short stature at the end of their cancer treatment. Growth failure was detected in 35 patients (13.8%, M:F = 15:20). The mean duration from a diagnosis of primary cancer to the development of growth failure was 3.9 ± 2.9 years (range, 1–12 years). Among them, 26 patients were characterized by stunted growth velocity within five years, and nine patients showed decreased growth velocity 8.1 ± 2.6 years after the primary cancer diagnosis (range 5–12 years). Two female patients, who were born as a prematurity and who were small for gestational age, consistently demonstrated short stature without decreased growth velocity. Therefore, they were not included in the growth failure.

Three of the four patients with syndromic disorders (BWS [n = 1], NS [n = 1], and WAGR syndrome [n = 1]) experienced stunted growth velocity during or after NCST treatment. The height-SDS decreased from -0.32 to -2.3 in the patient with BWS, from -1.51 to -2.99 in the patient with NS, and from 0.45 to -3.15 in the patient with WAGR syndrome.

The height-SDS of patients with growth failure was decreased from -0.25 ± 1.12 to -2.47± 0.77 at the final visit (*p* < 0.001). The average serum IGF-1 SDS of these patients with growth failure was -1.58 ± 1.14. Among the 35 patients with growth failure, GH provocation tests were performed in four patients who underwent head and neck RT. As a result, two patients were diagnosed with GHD: one female with isolated GHD and another female with multiple pituitary hormone deficiencies (MPHD). Five survivors had a stunted pubertal growth spurt due to hypergonadotropic hypogonadism. One patient developed short stature due to the prolonged use of prednisolone to treat chronic graft-versus-host disease (GVHD).

Seven patients (7/35, 20%) were treated with recombinant human growth hormone (rhGH) therapy. Two of these were diagnosed with GHD. The other five patients with significant growth delay experienced chronic renal disease caused by cancer treatment. The rhGH therapy was started after a mean duration of three years after completion of the NCST treatment. Chronological age at initiation of rhGH therapy was 10.1 ± 2.7 years (range, 7–14 years). Height-SDS (-2.72 ± 0.78) did not significantly improve after a mean 2.4 years of rhGH treatment (-2.28 ± 0.93, *p* = 0.128), although serum IGF-1 SDS increased from -1.72 ± 0.56 to 0.63 ± 2.04 (*p* = 0.018). Adverse effects were not seen with the rhGH therapy.

Factors that increased the risk of growth failure were female gender (HR = 2.70, *p* = 0.011), duration of cancer treatment between 25 and 36 months (HR = 6.36, *p* = 0.009), HSCT (HR = 2.58, *p* = 0.044), and RT (HR = 5.34, *p* < 0.001).

The final height of 12 of the 90 patients (13.3%) was below -2.0 SDS. To assess the factors that affected final height, univariable and multivariable linear regression analyses were performed in the 90 patients (M:F = 60:30) who reached final height (**Table 1 in Supplementary Material**). As a result, as treatment duration increased by a month, the final height-SDS decreased by -0.063 SDS (*B* = -0.063, *p* = 0.029). In addition, the final height decreased by -0.163 SDS when the summed AAD score increased by a point (*B* = -0.163, *p* = 0.028). The initial height-SDS was found to positively correlate with the final height-SDS (*B* = 0.996, *p* < 0.001).

#### 2. Obesity and Dyslipidemia

Obesity was observed in 18 patients (7.1%, M:F = 8:10). They were not obese at their initial cancer diagnosis, but developed obesity during or after NSCT treatment. The mean duration from initial cancer diagnosis to the development of primary hypothyroidism was 6.7 ± 4.3 years (range, 2-16 years). Multivariable Cox regression analysis revealed that female gender increased the risk of obesity (HR = 3.22, *p* = 0.047). In addition, HSCT increased the risk of obesity; however, this finding did not have statistical significance (HR = 3.30, *p* = 0.058).

Eight patients (3.2%, M:F = 4:4) were diagnosed with dyslipidemia. On average, it took 5.4 ± 5.0 years for dyslipidemia to present after the initial diagnosis of cancer (range, 2-17 years). Six of the patients were obese when they developed dyslipidemia. Five of them exhibited high low-density lipoprotein (LDL)-cholesterol levels (169.2 ± 10.1 mg/dL), and six patients showed hypertriglyceridemia (405.2 ± 155.0 mg/dL). Three exhibited mixed dyslipidemia with hypertriglyceridemia and hypercholesterolemia.

#### 3. Hypergonadotropic Hypogonadism and Precocious Puberty

At the last follow-up, 132 patients (52%) were over 13 years of age for females and 14 years of age for males. Of these, 16 patients (12.1%, M:F = 2:12) developed hypergonadotropic hypogonadism. The mean duration from initial cancer diagnosis to the development of gonadal dysfunction was 5.4 ± 3.8 years (range, 2–12 years). Female patients manifested primary ovarian failure 4.9 ± 3.7 years after initial cancer diagnosis, while male patients were diagnosed with gonadal dysfunction after 9.0 ± 2.8 years.

The mean LH and FSH levels of these patients at diagnosis of hypogonadism were 20.5 ± 18.7 mIU/mL (range, 5.5–72.4 mIU/mL) and 54.6 ± 37.1 mIU/mL (range, 5.9–132 mIU/mL), respectively. The mean levels of estradiol in females and testosterone in males were 5.4 ± 2.3 pg/mL (range, 4.0–11.0 pg/mL) and 4.6 ± 3.4 ng/mL, respectively. There were 14 patients (one male and 13 females) under sex hormone replacement therapy.

One female with facial rhabdomyosarcoma who underwent head and neck RT showed MPHD, including GHD, central hypothyroidism, adrenal insufficiency, and hypogonadotropic hypogonadism. A 15-year-old female with osteosarcoma manifested hypothalamic amenorrhea. Two patients with hepatoblastoma developed central precocious puberty (CPP) at seven years of age, which was three and five years after they completed cancer treatments.

Factors that increased the risk of gonadal failure in patients of post-pubertal age were female gender (HR = 30.31, *p* = 0.001), HSCT (HR = 46.39, *p* < 0.001), and RT (HR = 5.56, *p* = 0.019) using multivariable Cox regression analysis. The patients who developed hypergonadotropic hypogonadism underwent HSCT at 10.6 ± 3.7 years on average while those without hypogonadism underwent HSCT at 5.8 ± 4.8 years of age (*p* = 0.002).

#### 4. Primary Hypothyroidism

Primary hypothyroidism was observed in 12 survivors (4.7%, M:F = 5:7). The mean duration from initial cancer diagnosis to the development of primary hypothyroidism was 4.1 ± 2.9 years (range, 2–12 years). All these patients were treated with RT and HSCT and were on levothyroxine therapy. Eleven of the 12 patients (91.7%) with hypothyroidism were survivors of neuroblastoma; the remaining patient had Ewing sarcoma. Among the eight patients who underwent ^131^I-MIBG therapy, six (6/8, 75%) developed overt hypothyroidism and have been treated with levothyroxine.

#### 5. Vitamin D Deficiency and Osteoporosis

Vitamin D deficiency was detected in seven patients (2.8%, M:F = 2:5). The mean duration from initial cancer diagnosis to the development of primary hypothyroidism was 2.4 ± 1.7 years (range, 0.5–5 years). The mean 25OHD3 level was 8.31 ± 3.7 ng/mL. Two patients showed radiologically confirmed rickets. One patient demonstrated renal tubulopathy caused by chemotherapeutic agents, leading to hypophosphatemic rickets.

Osteoporosis was found in three of the 23 survivors who underwent a DEXA scan (13%, M:F = 1:2). On average, it took 2.3 ± 0.6 years for osteoporosis to develop after the initial NCST diagnosis (range, 2–3 years). The mean BMD *Z*-scores of the femur neck and L-spine were -3.1 ± 0.3 and -2.1 ± 1.2, respectively. One female patient with high-risk neuroblastoma had multiple hormonal deficiencies, including primary hypothyroidism, vitamin D deficiency, and hypergonadotropic hypogonadism. A male patient with osteoporosis had been treated with prednisolone for chronic GVHD following allogeneic HSCT. Another female with Ewing sarcoma had primary ovarian failure after autologous HSCT.

## Discussion

This study comprehensively analyzed endocrine complications in 253 childhood survivors of NCSTs. In this study, 30% of survivors developed at least one endocrine complication during the follow-up period, similar to a previous study on neuroblastoma (28%) and rhabdomyosarcoma of the head and neck (35%) (16). Shalitin et al. (18) described how 31.7% of survivors with NCSTs manifested at least one endocrine abnormality during a nine-year observation period, which is a comparable prevalence with our cohort.

In the present study, impaired growth was the most common endocrine complication after NCST treatment. Knijnenburg et al. (33) reported that 8.9% of 573 cancer survivors had a height ≤ -2 SDS at adulthood, which was slightly lower than the value obtained in our study. A treatment duration of between 25 and 36 months, HSCT, and exposure to RT were identified as the main risk factors for growth failure in the current study. Longer treatment duration implies tumor recurrence or metastasis that requires a more intensive treatment regimen, which, in turn, leads to impaired growth rate. The decrease in the risk of impaired growth in patients whose duration of treatment was ≥ 36 months was presumed to be due to a higher rate of mortality in these patients.

Growth failure in childhood NCST survivors was not associated with the hypothalamic-pituitary axis because only a few patients underwent head and neck RT, as in our cohort with two GHD patients. Multivariable linear regression analysis demonstrated that the summed AAD score negatively correlated with final height. Thus, it can be presumed that stunted growth velocity is caused by the direct skeletal toxicity of chemotherapeutic agents on the growth plate (17, 34). Doxorubicin inhibits chondrocyte destruction *in vitro* (35), which subsequently inhibits bone formation (34). Actinomycin D and purine antimetabolites reduced the proliferation of epiphyseal chondrocytes *in vitro* (36). Trans-retinoic acid induced premature epiphyseal closure in patients with high-risk neuroblastoma (37). Cyclophosphamide causes apoptosis in the proliferative zones of rat growth plates (38). In addition, a higher AAD score suggests an advanced primary cancer, which can impair general health, subsequently leading to growth failure. Decreased serum IGF-1 levels due to malnutrition during high-dose polychemotherapy and dexamethasone can also affect linear growth (34, 39). Another explanation for a decreased final height is the early onset of puberty or diminished pubertal growth spurt due to hypogonadism (40, 41).

Recent studies did not show a significant increase in the development of second neoplasms of the central nervous system in childhood cancer survivors who were treated with rhGH (42–45). Similarly, long-term follow-up data did not demonstrate a significant change in the risk of tumor recurrence or an increased risks of mortality in survivors treated with rhGH (46, 47). Traditionally, the recommendation has been that clinicians should start rhGH therapy for survivors of malignant tumors at least one year after the childhood cancer survivor is disease free (31). In our cohort, the mean duration for the commencement of rhGH therapy was three years after completion of NCST treatment. Of seven patients who were treated with rhGH, two patients were diagnosed with GHD, and the remaining five had chronic kidney disease (CKD). The efficacy of rhGH therapy in patients with CKD has previously been demonstrated (48). The efficacy of rhGH therapy could not be assessed in our cohort owing to the relatively short periods of rhGH treatment (a mean of 2.4 years) and the small number of patients.

The incidence of obesity in the current cohort (7.1%) was substantially higher than that in a previous study on NCST survivors (1.4%) (18). However, a much higher incidence (i.e., obesity of 31.7%) was reported in research on childhood cancer survivors (49). The prevalence of obesity in childhood survivors with acute lymphoblastic leukemia was shown to be substantially high (29%–69%) in one meta-analysis (50). The pathogenesis of dyslipidemia and obesity is mediated by factors related to anticancer treatment (chemotherapy, corticosteroids, and RT) and additional factors (i.e., genetic, dietary, or individual) (51). A high risk of obesity was associated with female gender and the initiation of cancer treatment at a young age (52). Thus, it is recommended that lipid profiles should be monitored in childhood cancer survivors (53).

Among patients who reached puberty, the prevalence of hypergonadotropic hypogonadism (12.1%) was higher than previous data on hematologic malignancies, lymphoma, and several extra-cranial solid tumors (6.3%) (54). As in previous research (54), the preservation of gonadal function was associated with a younger age at HSCT. In our study, we found significant difference in HSCT age between the two groups with or without hypogonadism, suggesting that gonadal function may be preserved if chemotherapy and/or HSCT are administered at a young age. The HR for primary gonadal dysfunction in female survivors was considerably high in our study. This difference in prevalence might be associated with underdiagnosed cases of male gonadal dysfunction and the peculiar amenorrhea symptoms in female patients. In males, Leydig cells were relatively resistant to chemotherapy, and the symptoms of Leydig cell failure tend to be subclinical in the majority of cases (30, 55). RT is a well-established cause of ovarian failure. TBI, rather than abdominal RT, was a risk factor for primary ovarian failure (56). In our cohort, exposure to RT and HSCT increased the risk of gonadal failure. RT has a direct harmful effect on oocytes, as even less than 4 Gy has been responsible for the death of 50% of human oocytes (57). Primary gonadal failure following HSCT is caused by conditioning regimens, including TBI and/or high dose chemotherapy (58). As gonadal insufficiency has a close association with HSCT, but not with a higher AAD score in our study, specific conditioning agents, such as busulfan or melphalan, may impair gonadal function rather than the total dose of alkylating agents. A previous research has demonstrated that a busulfan-based conditioning regimen has a higher risk of primary ovarian failure (59).

Primary hypothyroidism occurred in 9.9% of survivors in a previous study of NCSTs (18), which is higher than our study (4.7%). Previously reported risk factors of hypothyroidism include neck irradiation (60), HSCT, alkylating agents (18), and ^131^I-MIBG therapy (17, 61). Given that all patients with hypothyroidism had a history of RT during cancer treatment, hypothyroidism and radiotherapy were closely associated in our cohort.

Childhood cancer survivors face significant bone health disruptions (62). Besides the direct harmful effect of chemotherapeutic agents on bones (34), concomitant GHD, hypogonadism, and hyperthyroidism influence BMD deficits (22). Some chemotherapeutic agents, such as cyclophosphamide and cisplatin, cause renal damage and the dysregulation of calcium and vitamin D metabolism (63). As with the finding of the current study, in other research, the prevalence of vitamin D deficiency was high owing to poor sun exposure and an insufficient intake of vitamin D and calcium (64). The 25OHD3 levels of childhood cancer survivors must be monitored to prevent osteoporosis and fractures beyond the tumor site.

Although the risk of endocrine complications is significantly increased in cancer survivors, they are likely to be lost to follow-up if they do not have current health problems. Therefore, education on the late effects of cancer treatment should be emphasized when managing this at-risk population (65).

There were several limitations to this study. As this study was performed retrospectively, data on the cumulative dosages of RT or chemotherapy were missing. BMD and serum 25OHD3 levels were not screened in all subjects. Twenty seven percent of patients were followed-up less than 5 years, which might be too short duration to confirm the long-term consequences of cancer treatment. In addition, the primary tumors of this cohort were heterogeneous, which may hamper the unified analysis of endocrine outcomes. Finally, we did not consider staging and NCST relapses in the analyses.

In conclusion, this study analyzed the endocrine outcomes of 253 childhood NCST survivors. HSCT and high AAD scores increased the risk of developing at least one endocrine complication. Since high-dose myeloablative chemotherapy was mainly used as the pre-conditioning regimen prior to HSCT, alkylating agents rather than TBI was associated with endocrine complications. Growth failure was the most common endocrine complication. Factors affecting the final height were the initial height, treatment duration and cumulative dose of alkylating agents. HSCT was highly related to gonadal dysfunction. Lifelong surveillance for endocrine events is highly recommended in patients with NCSTs who were treated with high-dose polychemotherapy.

## Data Availability Statement

The original contributions presented in the study are included in the article/**Supplementary Material**. Further inquiries can be directed to the corresponding author.

## Author Contributions

YL and J-HC designed the research and wrote the manuscript. YL analyzed the data. JS, YC, HK, K-NK, HI, and H-WY collected the data. All authors contributed to the article and approved the submitted version.

## Funding

This research was supported by the Basic Science Research Program through the National Research Foundation of Korea (NRF), funded by the Ministry of Education (2017R1D1A1B03029638).

## Conflict of Interest

The authors declare that the research was conducted in the absence of any commercial or financial relationships that could be construed as a potential conflict of interest.
