# Supplementary material for: Endocrine Complications in Children and Adolescents With Non-Central Nervous System Solid Tumors
Source: Front Endocrinol (Lausanne). 2021 Mar 17;12:610730. doi: 10.3389/fendo.2021.610730 (PMC8011158; doi:10.3389/fendo.2021.610730)
Supplement: Supplementary file 1 [file Table_1.docx]

Supplementary Material

**Table 1** Univariable and multivariable linear regression analysis of determinants for final adult height-SDS.

| . | Univariable analysis | | | Multivariable analysis | | |
| --- | --- | --- | --- | --- | --- | --- |
| Variable | *B* | 95% CI | *p* value | *B* | 95% CI | *p* value |
| Age at cancer diagnosis (years) | 0.042 | -0.020, 0.103 | 0.182 |  |  |  |
| Initial height-SDS | 0.856 | 0.680, 1.033 | < 0.001 | 0.996 | 0.568, 1.423 | < 0.001 |
| Treatment duration (months) | -0.009 | -0.037, 0.019 | 0.512 | -0.063 | -0.119, -0.007 | 0.029 |
| Number of treatment modalities | -0.607 | -0.966, -0.247 | 0.001 |  |  |  |
| Summed AAD scores | -0.026 | -0.139, 0.087 | 0.650 | -0.163 | -0.307, -0.019 | 0.028 |
| Cumulative dose of RT (Gy) | -0.006 | -0.021, 0.008 | 0.398 |  |  |  |
| Mid-parental height (MPH) | 0.912 | -0.207, 2.030 | 0.105 |  |  |  |

CI: confidence interval; SDS: standard deviation scores; AAD: alkylating agent dose; RT: radiotherapy; Gy: gray.
